# Mutations in the *dwarf3* gene confer height stability in sorghum

**DOI:** 10.1002/tpg2.20466

**Published:** 2024-05-19

**Authors:** Elisabeth Diatta‐Holgate, Ben Bergsma, Mitchell R. Tuinstra

**Affiliations:** ^1^ Agronomy Department Purdue University West Lafayette Indiana USA

## Abstract

Dwarfism is a useful trait in many crop plants because it contributes to improved lodging resistance and harvest index. The mutant allele *dw_3_‐ref* (*dwarf_3_‐reference*) of sorghum [*Sorghum bicolor* (L.) *Moench*] is characterized by an 882 bp tandem duplication in the fifth exon of the gene that is unstable and reverts to wild‐type at a frequency greater than 0.001 in many genetic backgrounds. The goal of this research was to identify stable alleles of *dw_3_* (*dwarf3*) that could be backcrossed into elite parent lines to improve height stability of the crop. To discover new alleles of *dw_3_*, a panel consisting mostly of sorghum conversion lines (SC‐lines) was screened by polymerase chain reaction for the 882 bp tandem duplication in the fifth exon of *dw_3_‐ref*. Sanger sequencing was used to characterize the DNA sequence of this fragment in genotypes that did not contain the 882 bp tandem duplication. Sequence analysis identified three indel mutations, including an 82 bp deletion, a 6 bp duplication, and a 15 bp deletion in this region of the gene. Field trials of the donor genotypes with these new alleles indicated no wild‐type revertants of *dw_3_‐sd3* (*dwarf_3_‐stable dwarf*), *dw_3_‐sd4*, and *dw_3_‐sd5*. These alleles were backcrossed into Tx430. Field trials of backcross progeny (BC_2_F_4_) with the *dw_3_‐sd3*, *dw_3_‐sd4*, and *dw_3_‐sd5* alleles indicated no revertants. The plant height and flowering time characteristics of the backcross progeny were similar or slightly shorter and earlier than the recurrent parent. These findings demonstrate that *dw_3_‐sd3*, *dw_3_‐sd4*, and *dw_3_‐sd5* alleles will be useful in breeding for the stable dwarf trait.

## Core Ideas

- Dwarfism is a useful trait in many crop plants including sorghum.
- Sorghum dwarf stability is linked to naturally occurring mutations in the *dwarf3* gene.
- The new *dwarf3* alleles *dw_3_‐sd3*, *dw_3_‐sd4*, and *dw_3_‐sd5* confer height stability to sorghum plants.

**Abbreviations**
ABC: adenosine triphosphate‐binding cassette
ACRE: Purdue Agronomy Center for Research and Education
BME: beta‐mercaptoethanol
*br2*
: *brachytic2*
CTAB: cetyltrimethylammonium bromide
*dw_3_*
: *dwarf3*
*dw_3_‐ref*
: *dwarf_3_‐reference*
*dw_3_‐sd*
: *dwarf_3_‐stable dwarf*
EDTA: ethylenediaminetetraacetic acid
PCR: polymerase chain reaction
PGP: P‐glycoprotein

## 1 INTRODUCTION

Dwarfism is a useful trait in sorghum [*Sorghum bicolor* (L.) *Moench*] because it enables the use of mechanical harvesting, improves lodging resistance, and enables increased fertilizer‐use efficiency (Hedden, 2003; Khush, 2001). The *dw_3_* (*dwarf3*) gene of sorghum and *br2* (*brachytic2*) gene of maize (*Zea mays* L.) are orthologous. Previous studies have shown that the dwarf phenotypes associated with the recessive *dw_3_* and *br2* alleles are caused by mutations in a P‐glycoprotein (PGP), which functions as a polar auxin transporter (Multani et al., 2003). PGPs are encoded by multidrug‐resistant genes and are classified as adenosine triphosphate‐binding cassette (ABC) transporters. These proteins transport hydrophobic molecules out of the cell (Noh et al., 2001). Overexpression experiments in *Arabidopsis thaliana AtPGP1* resulted in longer hypocotyls in plants with gene overexpression and shorter hypocotyls when antisense DNA was over‐expressed (Noh et al., 2001; Sidler et al., 1998). These dwarf phenotypes were characterized by shorter internode lengths than wild‐type plants, caused by the lack of auxin stimulus required for cell elongation (Karper & Quinby, 1947; Multani et al., 2003).

The mutation in *dw_3_‐ref* (*dwarf_3_‐reference*) of sorghum is an 882 bp tandem duplication in exon 5, which causes the protein to be nonfunctional (Multani et al., 2003). This allele is known to be unstable with progeny of a homozygous *dw_3_‐ref* plant reverting to *Dw_3_* tall, wild‐type at a rate of around 1/600 plants, depending on the genetic background of the accession (Karper, 1932). Karper (1932) hypothesized that the reversion phenotype was caused by a mutation during gametogenesis, which Multani et al. (2003) confirmed as an unequal crossing‐over event during meiosis. In *dw_3_‐ref* sorghum, the gamete with the gene losing the duplication becomes *Dw_3_* wild type and exhibits a tall phenotype since the encoded auxin transporter can properly function (Multani et al., 2003).

The presence of height mutants in sorghum seed production is often managed through rouging, which increases labor and production costs and is not always reliable because environmental conditions such as drought can reduce plant height and make it difficult to differentiate height mutants from dwarf genotypes. Stable dwarfism ensures homogenous plant height and simplified mechanized harvesting. The development of stable height sorghum varieties or hybrids represents a solution to the problem.

A stable dwarf allele, *dw_3_‐sd2* (*dwarf_3_‐stable dwarf*), was previously identified in the sorghum line Tx2737, whose stable dwarf phenotype is due to a 6 bp deletion, which caused a two amino acid deletion in the translated protein (Barrero Farfan et al., 2012). This discovery pointed to the potential existence of different dwarf mutations in the *dw_3_* gene, some of which could be additional stable sources of the trait. In this study, a panel of dwarf‐stature sorghum accessions representing the genetic diversity of the crop was screened for novel *dw_3_* mutations. New *dw_3_* alleles with small insertions or deletions (indels) were identified and characterized for stability and impacts on plant height and maturity.

## 2 MATERIALS AND METHODS

### 2.1 Sorghum diversity panel

A sorghum diversity panel consisting of 300 sorghum breeding lines and sorghum conversion lines (SC‐lines) described by Casa et al. (2008) was screened by polymerase chain reaction (PCR) to discover novel *dw_3_* alleles. Fresh tissue samples were collected from each accession in the sorghum diversity panel by sampling a 5 cm segment of young leaf tissue in a 1.5 mL tube. The samples were processed by adding liquid nitrogen to each tube and grinding the leaf tissue with a small pestle to obtain a fine powder. DNA was extracted from each sample using a high throughput cetyltrimethylammonium bromide (CTAB) DNA extraction protocol (Dietrich et al., 2002). The CTAB buffer was made of 100 mM Tris pH 7.5, 0.7 M NaCl, 10 mM ethylenediaminetetraacetic acid (EDTA), 1% CTAB, and 0.17% beta‐mercaptoethanol (BME). First, 500 µL pre‐warmed buffer at 65°C was added to each sample tube, mixed, and incubated at 65°C for 30 min in a shaking water bath. The samples were thoroughly mixed during the incubation period, extracted with 500 µL of 24:1 chloroform/octanol mixture, and centrifuged at 14,000 × *g* for 10 min. A volume of 350 µL of the aqueous layer was transferred to a new tube, and 350 µL of cold (−20°C) isopropyl alcohol was added. After mixing gently to precipitate DNA, the samples were spun for 10 min at 14,000 × *g* to pellet the DNA. The pellets were washed with 500 µL 70% ethanol. After discarding the ethanol, the tubes were briefly centrifuged, and the residual ethanol was removed using a pipette. Pellets were rehydrated in 100 µL double‐distilled water and incubated on a heat block at 75°C for about 10 min to destroy residual DNase activity.

To extract DNA of the backcross progeny, a modified protocol combining some steps of the CTAB (Allen et al., 2006) and the mixed alkyl trimethyl ammonium bromide (MATAB) (Risterucci et al., 2000) procedure was used. Leaf samples were ground to a fine powder using a Qiagen tissue lyser at 30 m/s, to which 750 µL of extraction buffer solution (1.2 M NaCl, 100 mM Tris pH 8, 20 mM EDTA pH 8, 2% CTAB, and 0.1% BME) was added, followed by 30‐min incubation at 65°C in a water bath with gentle vortexing every 5 min. To each tube was added 750 µL of 24:1 chloroform:isoamyl alcohol. After mixing by continuous hand shaking for 1 min, the product was centrifuged at 13,000 × *g* for 20 min, then 600 µL of the supernatant was transferred to a new 1.5 mL Eppendorf tube and 600 µL of cold (−20°C) isopropyl alcohol was added to each tube. Tubes were gently shaken for 1 min, stored at −20°C for 2 h, centrifuged at 13,000 × *g* for 20 min, and the supernatant removed. The pellet was washed once in 500 µL of cold (−20°C) 70% ethanol and air dried. DNA was resuspended in HPLC grade water.

DNA concentration and quality were checked on a ThermoScientific Nanodrop 1000 Spectrophotometer, and samples with A_260/280_ above 1.8 were considered pure and diluted to 50 ng/µL prior to performing the PCR.

### 2.2 PCR screening for novel *dw_3_* alleles

The panel of 300 sorghum accessions was screened using PCR to amplify the 882 bp tandem duplication in *dw_3_*. The sorghum reference genome sequence obtained from Phytozome (Goodstein et al., 2012) was used to design the primers. The forward and reverse primers were labeled 4F (5′‐CGTCCTGCAGAAGATGTTCATGAAGG‐3′) and 5R (5′‐GTGCGCCACCACGATGGTGGTGC‐3′), respectively (Barrero Farfan et al., 2012). PCR was performed in a 25 µL reaction containing 1X MangoMix (Bioline USA Inc.), 0.4 µM of each primer, 0.125 µg of DNA, 1.25 µL dimethyl sulfoxide, and double‐distilled water. A PCR protocol with a touchdown step (Don et al., 1991) was used to amplify the locus, including an initial denaturation cycle at 95°C for 3 min, followed by 40 cycles of denaturation at 95°C for 45 s, annealing for 55 s, and one cycle of extension at 72°C for 2.5 min. The annealing temperature was set at 68°C for 10 cycles, stepping down 1°C every five cycles until reaching 65°C for the final 35 cycles. The PCR reactions were performed on a Bio‐Rad C1000 Thermal Cycler (Bio‐Rad Laboratories, Inc.), and the results were visualized on agarose gel made with 1% TAE buffer.

**Core Ideas**

- Dwarfism is a useful trait in many crop plants including sorghum.
- Sorghum dwarf stability is linked to naturally occurring mutations in the *dwarf3* gene.
- The new *dwarf3* alleles *dw_3_‐sd3*, *dw_3_‐sd4*, and *dw_3_‐sd5* confer height stability to sorghum plants.

### 2.3 DNA sequencing and sequence analysis

The amplified DNA samples from genotypes that did not include the 882 bp tandem duplication were purified using the ExoSAP‐IT Express PCR product cleanup reagent using the manufacturer's instructions (ThermoFisher.com). Sanger sequencing was performed at Azenta Life Sciences using the 4F primer (5′‐CGTCCTGCAGAAGATGTTCATGAAGG‐3′) (Barrero Farfan et al., 2012). Multiple sequence alignment of the samples was done with the *dw_3_* reference sequence of BTx623, *Sobic.007G163800.1*, via ClustalW multiple sequence alignment tool of BioEdit (Hall, 1999).

### 2.4 Dwarf allele introgression

**FIGURE 1:**
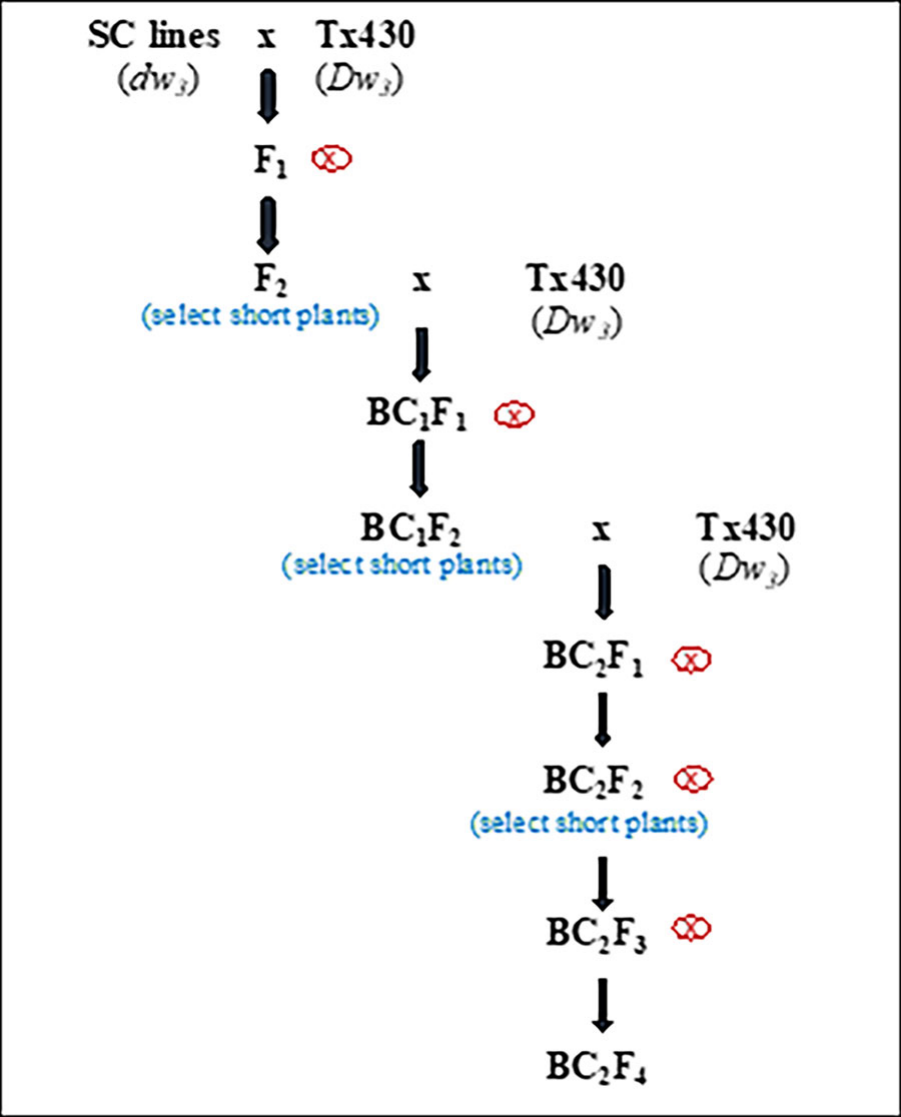
Backcross introgression of the dwarf alleles into Tx430. Three donor lines (SC124, SC134, and SC991) carrying the *dw_3_‐sd3, dw_3_‐sd4*, and *dw_3_‐sd5* alleles were crossed to Tx430 *Dw_3_*_._ The BC_2_F_4_ progeny are conversions of Tx430 carrying *dw_3_‐sd3*, *dw_3_‐sd4*, and *dw_3_‐sd5* alleles.

Tx430 carries the unstable *dw_3_‐ref* allele that can revert to wild‐type. A wild‐type revertant of Tx430 (*Dw_3_*), genetically similar to Tx430 *dw_3_‐ref* except for the mutation, was identified and used as a male parent in developing the backcross populations (Figure 1). Tx430 *Dw_3_* was crossed as a male to sorghum lines carrying unique *dw_3_* alleles. The F1 progeny from each cross were self‐pollinated, and the F_2_ seeds were planted in plots to verify 3:1 segregation for the *Dw_3_* allele. The short plants from each row carrying the *dw_3_* alleles were backcrossed to Tx430 *Dw_3_*, and the resulting BC_1_F_1_ plants were self‐pollinated to obtain BC_1_F_2_ populations. Short plants in each BC_1_F_2_ family were backcrossed to Tx430 *Dw_3_* and the resulting BC_2_F_1_ plants were self‐pollinated to obtain BC_2_F_2_ families. The *dw_3_* plants in each family were self‐pollinated for two generations to generate an array of BC_2_F_4_ progeny for each of the new *dw_3_* alleles (Figure 1).

### 2.5 Stability performance of the new *dwarf3* alleles

The stability of each new *dw_3_* allele was assessed in field trials at the Purdue Agronomy Center for Research and Education (ACRE) in 2020. Each of the donor lines and the recurrent parent (Tx430, SC124, SC134, and SC991) were planted in an experiment using a randomized complete block design with four replicates. Each plot was planted as a 91.4‐m single‐row plot with 0.76 m between plots. The number of plants for each plot was recorded and the frequency of *Dw3* plants was calculated as the ratio of tall plants over the total number of plants recorded for the genotype. *Dw3* plants are visually significantly taller than the dwarf *dw3* entries, hence they are easily identified in the field.

The new *dw_3_* alleles from SC124, SC134, and SC991 were backcrossed into Tx430. Nine backcross progeny were advanced to BC_2_F_4_ for each new allele and assessed in field trials at the ACRE in 2020 using a randomized complete block design with three replicates. Each plot was planted as a 91.4‐m single‐row plot. The number of plants for each plot was recorded, and the frequency of *Dw3* plants was calculated as the ratio of tall plants over the total number of plants recorded for the genotype.

Based on the overall performance of the backcross progeny in 2020, the 10 best BC_2_F_5_ lines were selected and planted at ACRE in 2022 along with their parents. Each BC_2_F_5_ line was planted in two replications with each replication planted as a 3.04‐m single‐row plot with 0.76 m between plots. Five random plants from each plot were randomly tagged to determine the average flowering time based on days from planting to 50% flowering and plant height measured as apex height.

## 3 RESULTS

### 3.1 Allele screening and description of the new dwarf alleles

**FIGURE 2:**
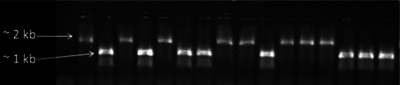
PCR products from a screen of *Sobic.007G163800* (*dwarf3)* in the sorghum diversity panel. The PCR fragments were visualized in a 1% agarose gel stained with ethidium bromide. The genotypes carrying the *dw_3_* reference allele produced PCR products (2006 bp) that are nearly two times longer than the WT (1124 bp) or new *dw_3_* alleles without the tandem duplication (1042, 1109, and 1130 bp).

PCR analyses were used to discover new *dw_3_* alleles in the sorghum diversity panel. The PCR product was 2006 bp long when the exon contained the tandem duplication (*dw_3_‐ref*) and 1124 bp long when the product contained no duplication (Figure 2). PCR products examined on 1% agarose gel revealed that 181 of the 300 accessions had a 2 kb band, which were assumed to be genotypes containing the *dw_3_‐ref* allele with the 882 bp duplication (Figure 2). All other lines exhibited a roughly 1 kb band that could indicate either wild‐type *Dw_3_* plants or novel *dw_3_* alleles. Therefore, all the samples with the 1 kb band were Sanger sequenced.

**FIGURE 3:**
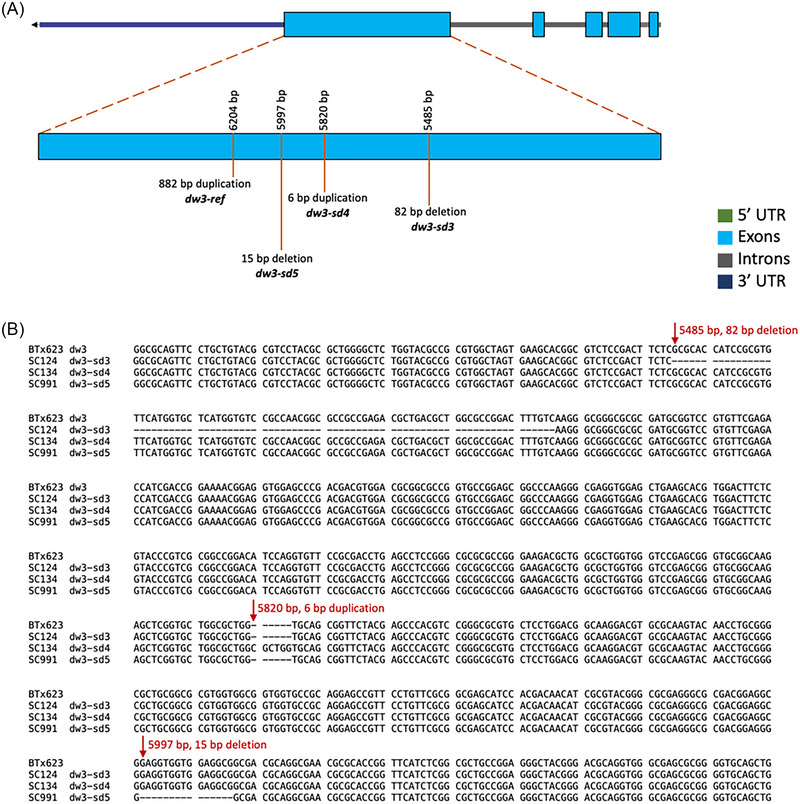
(A) Gene scheme of *Sobic.007G163800* showing the insertions and deletions found in the sorghum diversity panel. The indels are located on exon 5 from Sorghum *bicolor* v3.1.1. (B) Sequence alignment of the region of exon 5 of *Sobic.007G163800* amplified from SC124, SC134, and SC991 compared to the reference genome, BTx623. The positions of the *dwarf3* indels are shown in red for the *dw_3_‐sd3* allele (82 bp deletion), *dw_3_‐sd4* allele (6 bp duplication), and *dw_3_‐sd5* allele (15 bp deletion). Positions do not include the untranslated regions (UTRs).

Sanger sequencing and sequence alignment of the accessions that had a 1 kb PCR fragment confirmed that those genotypes did not have the 882 bp duplication in the fifth exon; however, many accessions carried additional mutations (Figure 3). Unique indels were identified in the population, including one insertion and two deletions (Table 1). The newly identified alleles are *dw_3_‐sd3* (82 bp deletion), *dw_3_‐sd4* (6 bp duplication), and *dw_3_‐sd5* (15 bp deletion) (Table 1).

**TABLE 1:** Description of new *dwarf3* alleles discovered in the sorghum diversity panel.

Mutations	Positions (bp)	PCR fragment size (bp)	Allele name	Source accessions
882 bp duplication^a^	6204–7035	2006	*dw_3_‐ref*	BTx623
82 bp deletion	5485–5566	1042	*dw_3_‐sd3*	SC124
6 bp duplication	5820–5825	1130	*dw_3_‐sd4*	SC134, SC1017, SC1038, SC1154, SC1155
15 bp deletion	5997–6011	1109	*dw_3_‐sd5*	SC991

Abbreviation: PCR, polymerase chain reaction.

^a^ Mutation previously discovered. All sequence positions are derived from the genomic sequence of *Sobic.007G163800* from version 3.1.1 of *Sorghum bicolor* which carries the 882 bp tandem duplication.

### 3.2 Stability performance

**FIGURE 4:**
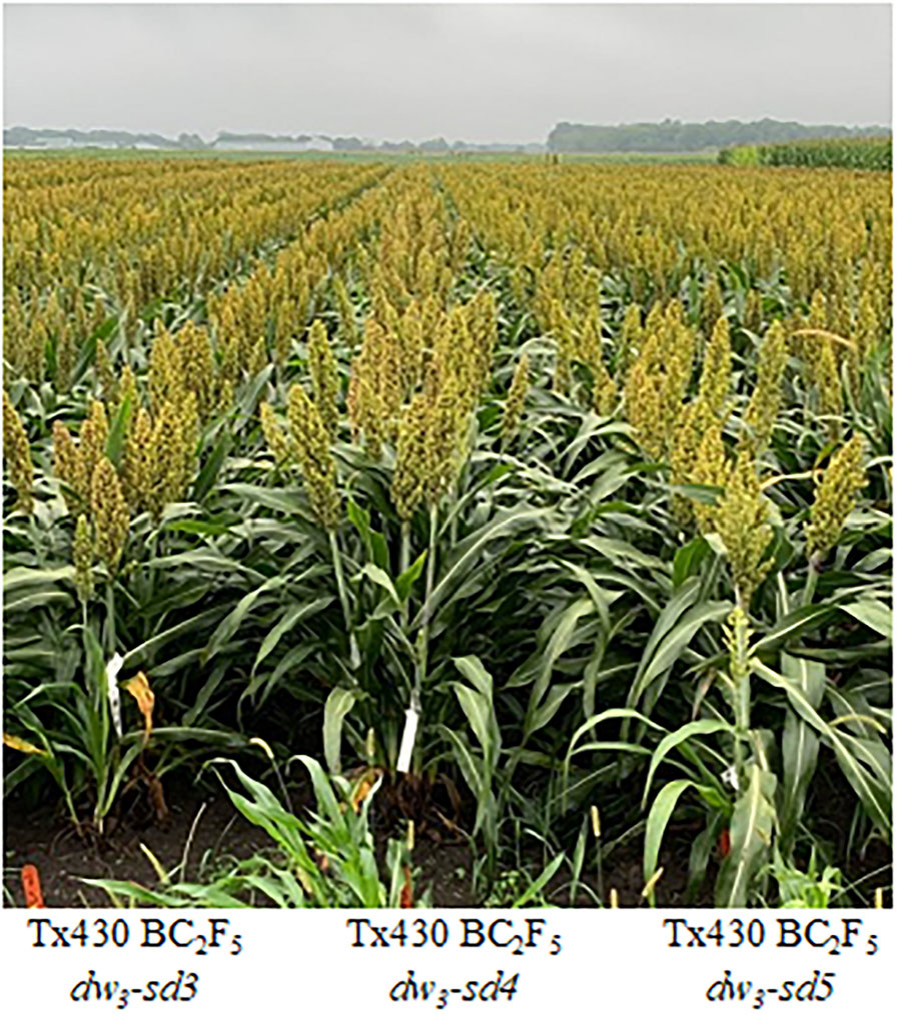
Sorghum trials of backcross progeny (BC_2_F_5_) of Tx430 with *dw_3_‐sd3*, *dw_3_‐sd4*, and *dw_3_‐sd5* in West Lafayette, IN, in 2020.

Field trials conducted to examine the stability of the three new *dw_3_* alleles revealed that *dw_3_‐sd3* (82 bp deletion), *dw_3_‐sd4* (6 bp duplication), and *dw_3_‐sd5* (15 bp deletion) were stable (Figure 4), while *dw_3_‐ref* exhibited a reversion rate for plant height under field conditions on average one in 300 plants (Table 2; Table S1). The backcross conversions of *dw_3_‐sd3*, *dw_3_‐sd4*, and *dw_3_‐sd5* in the Tx430 genetic background were also shown to be stable with no mutants observed in 14,601, 16,685, and 14,822 plants, respectively (Table 3; Table S2). The average flowering times of selected BC_2_F_5_ progeny were between 69.6 and 71.4 days with individual progeny being similar or slightly earlier than Tx430 (Table 4; Table S3). The average plant heights of BC_2_F_5_ progeny were between 102 and 112 cm with individual progeny being similar to or slightly shorter than Tx430 (Table 4).

**TABLE 2:** Plant height stability of indel donor lines.

Genotype	Allele	Total number of plants	Number of *Dw_3_* mutants	Frequency of *Dw_3_* plants
Tx430	*dw_3_‐ref*	1095	4	0.365
SC124	*dw_3_‐sd3*	2734	0	0
SC134	*dw_3_‐sd4*	2651	0	0
SC991	*dw_3_‐sd5*	3468	0	0

**TABLE 3:** Plant height stability of backcross progeny.

Pedigree	Allele	Generation	Total number of plants	Number of *Dw_3_* mutants	Frequency of *Dw_3_* plants
SC124/Tx430//Tx430///Tx430	*dw_3_‐sd3*	BC_2_F_4_	14,601	0	0
SC134/Tx430//Tx430///Tx430	*dw_3_‐sd4*	BC_2_F_4_	16,685	0	0
SC991/Tx430//Tx430///Tx430	*dw_3_‐sd5*	BC_2_F_4_	14,822	0	0

**TABLE 4:** Variation in flowering time and plant height among backcross progeny with *dw_3_‐sd3*, *dw_3_‐sd4*, and *dw_3_‐sd5*.

			Flowering (days after planting)		Plant height (cm)	
Pedigree	Allele	Generation	Mean	Standard deviation	Mean	Standard deviation
SC124/Tx430//Tx430///Tx430	*dw_3_‐sd3*	BC_2_F_5_	71.4	0.3	112	4.1
SC134/Tx430//Tx430///Tx430	*dw_3_‐sd4*	BC_2_F_5_	69.7	1.0	104	2.5
SC991/Tx430//Tx430///Tx430	*dw_3_‐sd5*	BC_2_F_5_	69.6	2.9	102	1.7
Tx430 *Dw_3_*	*Dw3*	BC_2_F_5_	70.7	1.3	203	6.4
Tx430 *dw_3_*	*dw_3_‐ref*	L	75.8	1.5	113	4.6

## 4 DISCUSSION

### 4.1 Dwarfism is a useful trait in sorghum

Selective breeding for advantageous plant traits can increase their value by improving the efficiency of how they are grown and harvested. In the case of sorghum, dwarf plants are useful because they generally exhibit improved resistance to lodging and are easier to mechanically harvest (Karper & Quinby, 1947). Due to the potential value of the dwarfing trait, it is useful to understand the natural diversity of the *dw_3_* locus. Identifying multiple sources of a desirable allele could be useful for introgression projects since the linkage drag from different genetic backgrounds may improve heterosis when derived lines are used in hybrid seed production. A survey of the diversity of a trait at a given locus can offer insights into past breeding methods and may reveal important gene regions, as indicated by high sequence conservation.

### 4.2 Sorghum dwarf height stability is linked to naturally occurring mutations

Multani et al. (2003) noted that new dwarfing alleles could be used to address the instability of the tandem duplication of *dw3‐ref* in sorghum. In this research, three new, stable dwarfing mutations were discovered in the *dw_3_* gene. Deletions and insertions have good potential for use in developing new stable dwarf phenotypes due to their likely disruption of protein function. The goal of this research was to discover new stable *dw_3_* alleles in diverse genetic backgrounds to improve the diversity of this region of Chromosome 7 in sorghum.

Novel *dw_3_* alleles containing insertions/deletions were identified in the sorghum diversity panel. The newly identified indels included an 82 bp deletion discovered in SC124, a 6 bp insertion in SC134, and a 15 bp deletion in SC991 for which the alleles were named *dw_3_‐sd3*, *dw_3_‐sd4*, and *dw_3_‐sd5*, respectively. The 82 bp deletion (*dw_3_‐sd3*) caused a frame shift in the DNA code, which led to the introduction of an early stop codon at position 963 in the peptide chain of SC124 resulting in the rest of the fifth exon not being translated. The 6 bp insertion (*dw_3_‐sd4*) led to the amino acids A1036 and L1037 added to the peptide chain without affecting the reading frame. The 15 bp deletion in *dw_3_‐sd5* led to five amino acids being deleted from the peptide chain with no additional disruptions observed in the amino acid sequence.

### 4.3 Dwarf stability

Field trials demonstrated that *dw_3_‐sd3*, *dw_3_‐sd4*, and *dw_3_‐sd5* alleles were dwarf‐stable. A study of backcross progeny with more than 14,000 plants representing each of these new alleles demonstrated that no revertant plants were found in any backcross lines that contained the *dw_3_‐sd3*, *dw_3_‐sd4*, and *dw_3_‐sd5* alleles, whereas the check with the reference allele (Tx430 *dw3‐ref*) had a revertant rate of about one in 270 plants, similar to what was reported by Barrero Farfan et al. (2012).

The phenological characteristics of the backcross progeny indicated that many of the lines were similar or slightly earlier in flowering time and similar or shorter in plant height compared to the recurrent parent Tx430. Given the importance of early maturity and short plant stature in sorghum production, this is a positive outcome that may add value to these lines as trait‐donors (Karper & Quinby, 1947).

Utilizing dwarf‐stable parent lines in seed production is desirable because of the reduced need for rouging in seed production fields. Rouging describes the process of manually removing unwanted height mutants and other off‐types from fields prior to pollen shed. When unfavorable weather or staffing challenges cause delays in rouging, any tall mutant plants can shed wild‐type pollen and substantially increase the frequency of off‐types in the next generation. If the frequency of height mutants is high enough, these seed lots may be discarded, thereby increasing the cost of these products. The commercialization of dwarf‐stable parent lines and hybrids greatly reduces this risk. Farmers also prefer the appearance of dwarf stable sorghum hybrids because these products are more uniform in production fields.

### 4.4 Concluding remarks

These studies suggest that *dw_3_‐sd3*, *dw_3_‐sd4*, and *dw_3_‐sd5* will be useful in breeding for a stable dwarf phenotype without negatively altering other agronomic characteristics.

## AUTHOR CONTRIBUTIONS

**Elisabeth Diatta‐Holgate**: Data curation; formal analysis; investigation; validation; visualization; writing—original draft. **Ben Bergsma**: Data curation; formal analysis; investigation; methodology; writing—original draft. **Mitchell R. Tuinstra**: Conceptualization; data curation; formal analysis; funding acquisition; investigation; methodology; project administration; resources; supervision; writing—review and editing.

## CONFLICT OF INTEREST STATEMENT

The authors declare no conflicts of interest.

## Supporting information

Supplementary Table S1. Plant height stability trial data of R.Tx430 and indel donors.

Supplementary Table S2. Plant height stability trial of backcross progeny.

Supplementary Table S3. Plant height and flowering time data of backcross progeny

## Data Availability

Field trial data for plant height stability and flowering time of donor and recurrent parents and backcross progenies are made available in Tables S1–S3.

## References

[tpg220466-bib-0001] Allen, G. C. , Flores‐Vergara, M. A. , Krasynanski, S. , Kumar, S. , & Thompson, W. F. (2006). A modified protocol for rapid DNA isolation from plant tissues using cetyltrimethylammonium bromide. Nature protocols, 1(5), 2320–2325. 10.1038/nprot.2006.384 17406474

[tpg220466-bib-0002] Barrero Farfan, I. D. , Bergsma, B. R. , Johal, G. , & Tuinstra, M. R. (2012). A Stable *dw3* allele in sorghum and a molecular marker to facilitate selection. Crop Science, 52(5), 2063–2069. 10.2135/cropsci2011.12.0631

[tpg220466-bib-0003] Casa, A. M. , Pressoir, G. , Brown, P. J. , Mitchell, S. E. , Rooney, W. L. , Tuinstra, M. R. , Franks, C. D. , & Kresovich, S. (2008). Community resources and strategies for association mapping in sorghum. Crop Science, 48(1), 30–40. 10.2135/cropsci2007.02.0080

[tpg220466-bib-0004] Dietrich, C. R. , Cui, F. , Packila, M. L. , Li, J. , Ashlock, D. A. , Nikolau, B. J. , & Schnable, P. S. (2002). Maize *Mu* transposons are targeted to the 5′ untranslated region of the *gl8* gene and sequences flanking *Mu* target‐site duplications exhibit nonrandom nucleotide composition throughout the genome. Genetics, 160(2), 697–716. 10.1093/genetics/160.2.697 11861572 PMC1461997

[tpg220466-bib-0005] Don, R. H. , Cox, P. T. , Wainwright, B. J. , Baker, K. , & Mattick, J. S. (1991). “Touchdown” PCR to circumvent spurious priming during gene amplification. Nucleic Acids Research, 19(14), 4008. 1861999 10.1093/nar/19.14.4008 PMC328507

[tpg220466-bib-0006] Goodstein, D. M. , Shu, S. , Howson, R. , Neupane, R. , Hayes, R. D. , Fazo, J. , Mitros, T. , Dirks, W. , Hellsten, U. , Putnam, N. , & Rokhsar, D. S. (2012). Phytozome: A comparative platform for green plant genomics. Nucleic Acids Research, 40(D1), D1178–D1186. 10.1093/nar/gkr944 22110026 PMC3245001

[tpg220466-bib-0007] Hall, T. A. (1999). BioEdit: A user‐friendly biological sequence alignment editor and analysis program for Windows 95/98/NT. Nucleic Acids Symposium Series, 41, 95–98.

[tpg220466-bib-0008] Hedden, P. (2003). The genes of the green revolution. Trends in Genetics, 19(1), 5–9. 10.1016/S0168-9525(02)00009-4 12493241

[tpg220466-bib-0009] Karper, R. E. (1932). A dominant mutation of frequent recurrence in sorghum. The American Naturalist, 66(707), 511–529. 10.1086/280457

[tpg220466-bib-0010] Karper, R. E. , & Quinby, J. R. (1947). Sorghum—Its production, utilization and breeding. Economic Botany, 1(4), 355–371. 10.1007/BF02858895

[tpg220466-bib-0011] Khush, G. S. (2001). Green revolution: The way forward. Nature Reviews Genetics, 2(10), 815–822. 10.1038/35093585 11584298

[tpg220466-bib-0012] Multani, D. S. , Briggs, S. P. , Chamberlin, M. A. , Blakeslee, J. J. , Murphy, A. S. , & Johal, G. S. (2003). Loss of an MDR transporter in compact stalks of maize *br2* and sorghum *dw3* mutants. Science, 302(5642), 81–84. 10.1126/science.1086072 14526073

[tpg220466-bib-0013] Noh, B. , Murphy, A. S. , & Spalding, E. P. (2001). *Multidrug Resistance*‐like genes of *Arabidopsis* required for auxin transport and auxin‐mediated development. The Plant Cell, 13(11), 2441–2454. 10.1105/tpc.010350 11701880 PMC139463

[tpg220466-bib-0014] Risterucci, A. M. , Grivet, L. , N'Goran, J. A. , Pieretti, I. , Flament, M. H. , & Lanaud, C. (2000). A high‐density linkage map of *Theobroma cacao* L. Theoretical and Applied Genetics, 101, 948–955. 10.1007/s001220051566

[tpg220466-bib-0015] Sidler, M. , Hassa, P. , Hasan, S. , Ringli, C. , & Dudler, R. (1998). Involvement of an ABC transporter in a developmental pathway regulating hypocotyl cell elongation in the light. The Plant Cell, 10, 1623–1636. 10.1105/tpc.10.10.1623 9761790 PMC144355

